# Pilot study exploring artificial intelligence for facial-image-based diagnosis of Marfan syndrome

**DOI:** 10.1016/j.heliyon.2024.e33858

**Published:** 2024-06-28

**Authors:** Danny Saksenberg, Sandip Mukherjee, Mohammad A. Zafar, Bulat Ziganshin, John A. Elefteriades

**Affiliations:** a Yale University School of Medicine, New Haven, CT, USA; b Emerge, Johannesberg, SA, USA

**Keywords:** Marfan syndrome, Facial image, Artificial intelligence, Connective tissue disease

## Abstract

**Background:**

Marfan Syndrome (MFS), a genetic disorder impacting connective tissue, manifests in a wide array of phenotypes which can affect numerous bodily systems, especially the thoracic aorta. The syndrome often presents distinct facial features that potentially allow for diagnostic clinical recognition. Herein, we explore the potential of Artificial Intelligence (AI) in diagnosing Marfan syndrome from ordinary facial images, as assessed by overall accuracy, F1 score, and area under the ROC curve.

**Methods:**

This study explores the utilization of Convolutional Neural Networks (CNN) for MFS identification through facial images, offering a novel, non-invasive, automated, and computerized diagnostic approach. The research examines the accuracy of Neural Networks in the diagnosis of Marfan Disease from ordinary on-line facial images. The model was trained on 80 % of 672 facial images (182 Marfan and 490 control). The other 20 % of images were used as the test set.

**Results:**

Overall accuracy was 98.5 % (0 % false positive, 2 % false negative). F1 score was 97 % for Marfan facies and 99 % for non-Marfan facies. Area under the ROC curve was 100 %.

**Conclusion:**

An Artificial Intelligence (AI) program was able to distinguish Marfan from non-Marfan facial images (from ordinary on-line photographs) with an extremely high degree of accuracy. Clinical usefulness of this program is anticipated. However, due to the limited and preliminary nature of this work, this should be viewed as only a pilot study.

## Highlights

• We explore Artificial Intelligence for detection of Marfan Disease (MFS) from simple facial images.
• AI proves extremely effective at detection of MFS.
• Overall accuracy is 98.5 % (0 false positives, 2 % false negatives).
• Clinical usefulness is anticipated.

In 1896, Antoine Marfan first reported the syndrome that bears his name in the Bulletin of the Medical Society of Paris. He described the physical features of Gabrielle, a six-year-old girl with long, thin extremities [1]. It has since been questioned whether that child actually suffered from Marfan syndrome or from a related disease (congenital contractural arachnodactyly) [2].

For the ensuing years, the diagnosis of Marfan's disease has been predicated on clinical judgment, based on a variety of physical features. “Experts” felt that they could identify Marfan's disease with a glance and confirm the diagnosis upon closer overall clinical evaluation. In 1996, the Ghent Nosology for clinical diagnosis of Marfan's Disease was articulated [3]. This advance identified specific features in various organ systems, which were then graded to yield numerical confirmation of the diagnosis of Marfan's disease.

Marfan syndrome has an incidence of approximately 1 in 3000–5000 human beings [4,5].

Caused by mutations in the FBN1 gene responsible for fibrillin-1 production, a protein essential to connective tissue, Marfan Syndrome exhibits a broad phenotypic range [6,7]. Recognizable physical features include disproportionately long limbs, arachnodactyly (long fingers and toes), tall stature, and distinct facial features like malar hypoplasia (underdeveloped cheekbones), dolichocephaly (elongation of the head), down-slanting palpebral fissures (elliptical opening between the two eyelids slants downward laterally), and retrognathia (recessed lower jaw). These unique physical manifestations present an opportunity to explore non-invasive diagnostic methods, such as facial image analysis.

In recent years, Artificial Intelligence (AI) has made a dramatic impact in clinical medicine [8]. For example, at many medical centers, the diagnosis of aortic dissection is first made by AI [9]. When AI reads a computerized tomographic scan (CT) as showing an aortic dissection, an urgent message is sent electronically to a battery of key team members—often before a radiologist has even seen the images. Via that notification, the operating room team can be mobilized for immediate surgical intervention. It has been shown that the accuracy of AI in this diagnosis (aortic dissection) is extremely high. Humans cannot be sure what features AI is using in making its immediate diagnosis of aortic dissection.

Some examples of the broad applicability of AI in general, and CNNs specifically, in medical imaging include: AiDoc (https://www.aidoc.com/)– a growing ecosystem of AI-enabled tools, currently encompassing diagnosis and management of several cardiovascular, neurologic, and radiology applications; AliveCor (https://alivecor.com/)- AliveCor has received FDA clearance for the use of AI to interpret ECGs to make determinations of multiple cardiac conditions, including sinus rhythm with premature ventricular contractions (PVCs), sinus rhythm with supraventricular ectopy (SVE), and sinus rhythm with wide QRS; Face2Gene (https://www.face2gene.com/)– a suite of phenotyping applications that facilitate comprehensive and precise genetic evaluations.

Convolutional Neural Networks (CNNs) are a type of deep learning model that excels in image analysis and recognition tasks [10]. Unlike traditional machine learning models, CNNs autonomously learn hierarchical representations from raw input data, eliminating the need for manual feature extraction. They consist of multiple layers, including convolutional layers for feature extraction, pooling layers for down-sampling data, and fully connected layers for final output predictions. CNNs have been effectively employed in a broad spectrum of applications, from autonomous vehicles to medical imaging diagnostics, showcasing their robust versatility [11].

We wondered if AI could accurately make the diagnosis of Marfan's disease based on facial features alone. We report herein our findings on this question.

## 1 Methods

*Data acquisition and preprocessing*. For the purpose of this study, a diverse dataset of facial images was assembled from several public sources: For non-Marfan faces, faces were excerpted from a public database of human faces provided by Flickr. For Marfan faces, faces were excerpted from pictures and videos relating to Marfan Syndrome primarily from the following sources: the Marfan Foundation (https://marfan.org/), the Marfan Trust (https://www.marfantrust.org), You Tube, Standalone articles.

In our data assembly process, we included any organization that dealt with Marfan Syndrome, including the Marfan Foundation, the Marfan Trust, and similar organizations. We methodically worked through all their sites' subpages for images identifying someone with MFS. Screenshots of the relevant faces on those pages and articles were taken.

We searched for “Marfan syndrome” and “Marfan syndrome faces” on Google and interrogated all the results, which likely contained images of people with MFS. We acquired screenshots where the context article clarified that the people in question had MFS.

Finally, we searched for videos on YouTube related to MFS, including medical videos discussing MFS and “living with MFS"-type videos and screen-snipped people's faces where the context made it clear that they had MFS. As for racial/ethnic breakdown, the dataset consisted of 83 % Caucasian/Hispanic subjects, 10 % black subjects, and 7 % Asian subjects.

Non-Marfan images: The Flickr-Faces-HQ public database of human faces was utilized to obtain images of individuals not diagnosed with Marfan Syndrome. Given the low incidence rate of Marfan Syndrome, any mislabeled images within the Flickr database were considered to have a negligible impact on the study. The total number of non-Marfan patient images used was 490, notwithstanding that several hundred thousand sample images are available from this dataset.

Inclusion and exclusion criteria: The inclusion criteria for the study were facial images of individuals either diagnosed with Marfan Syndrome (as described above) or those assumed not to have the syndrome (Flickr database). Images were required to clearly show the front of the face, to be free from obscuring facial coverings (although images where the subject was wearing glasses or goggles were not removed), and to be of adequate quality and resolution. Images were excluded if they failed to meet these criteria. No age limits were imposed, and the datasets include images of juveniles.

The images were preprocessed, normalized to a uniform size, and augmented (reconfigured) to increase the dataset's size and diversity. The images were preprocessed using the TensorFlow library within a Python environment. This preprocessing included rescaling the pixel-intensity values to a value between 0 and 1, and resizing each image so that they would all have a uniform height of 450 pixels and uniform width of 250 pixels. Their backgrounds were also removed (converted to blank pixels) to ensure the model was learning from the facial features only and not from anything in the background.

The images were also augmented (reconfigured) to increase the dataset's size and diversity. This is done by applying various transformations to the original images, creating multiple different versions of each image. In particular, the following augmentation techniques were applied.

1) rotation – images are rotated by a random angle
2) zooming – randomly zooming into or out of an image
3) brightness, contrast and saturation adjustment – randomly changing the brightness, contrast and saturation of an image
4) noise addition – adding random noise to the image.

The following paragraphs describe the pattern-recognition methods used in this work. In this study, a Convolutional Neural Network (CNN), a prominent architecture in deep learning, was used to identify and recognize the facial features relevant to the diagnosis Marfan Syndrome from facial images. CNNs diverge from traditional image analysis methods in their approach to processing and learning from image data.

CNN's were chosen for this task because of their ability to recognize and learn useful features within images without requiring explicit guidance. This autonomous feature extraction capability significantly reduces the need for manual feature engineering, making CNNs highly efficient and effective in various applications, including medical image analysis [12].

Moreover, CNNs are designed to be invariant to translations and rotations of the input image, meaning they can recognize features regardless of their position or orientation within the image. This is achieved through the use of convolutional and pooling layers, which allow the network to detect local patterns and spatial hierarchies. As a result, once a CNN learns a specific facial feature indicative of Marfan Syndrome, it can identify this feature in new images, even if the feature appears in different locations or orientations [13].

Additionally, it is important to note that the features identified by CNNs may differ from those used by humans. CNNs often focus on local patterns and textures, which might not be the primary focus of human visual analysis. This difference highlights the unique way in which CNNs learn and apply heuristics, often leading to improved performance on specific tasks by leveraging different aspects of the visual data compared to human observers [12].

In the context of our study, these high-level features could correspond to combinations of multiple facial characteristics distinctive of Marfan Syndrome, aligning with prior studies that underscore the value of assessing a combination of facial traits.

The CNN model was trained on the preprocessed images, with each image labeled as ‘Marfan’ or ‘Non-Marfan’. The images were fed into the network, which then learned to identify and abstract relevant facial features associated with Marfan Syndrome, ultimately enabling it to classify unseen images effectively as “Marfan” or “non-Marfan”.

To assess the model's performance, a variety of metrics were calculated. The model was trained using a random sample of 80 % of the images in the dataset (“the training dataset”). The remaining 20 % were held back to be used as unseen data on which to assess the model performance (“the validation dataset”). The statistical analysis for this study was performed using Python with the Pandas and scikit-learn libraries. The following metrics were calculated to evaluate the performance of our model in classifying Marfan and Non-Marfan cases.

**Fig. 1:**
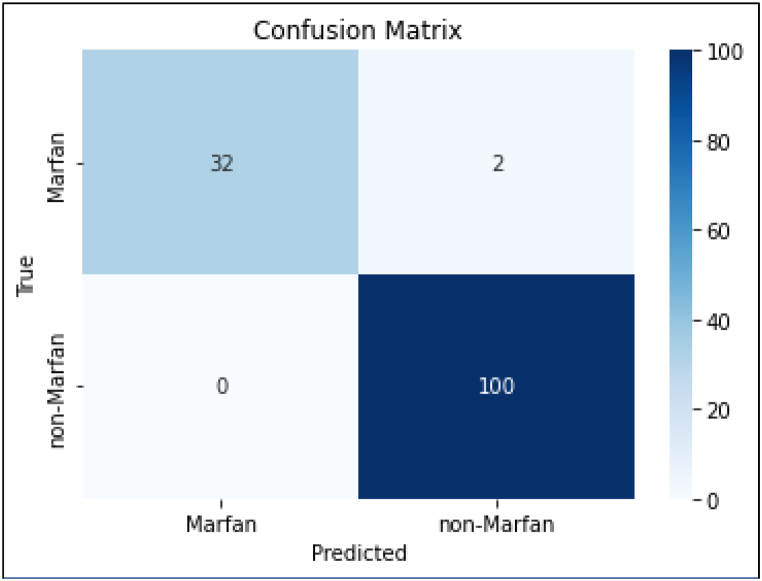
Confusion matrix. Validation set metrics.

**Fig. 2:**
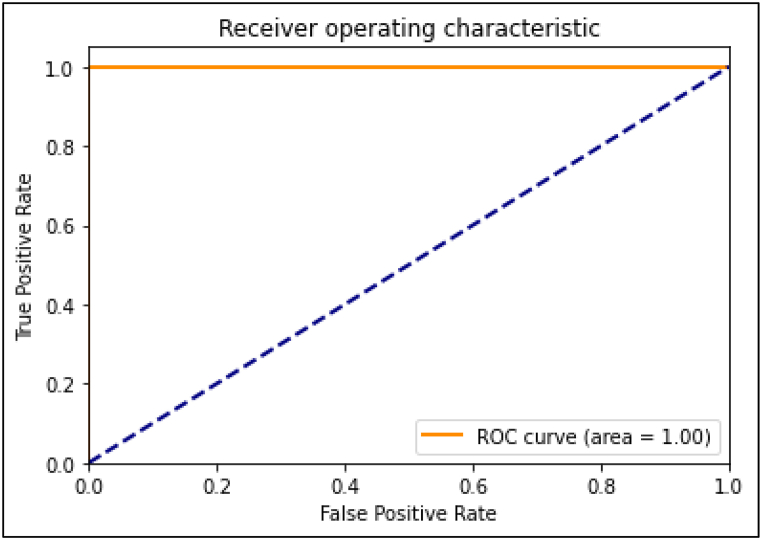
Validation set ROC curve. The area under the curve is 100 %.

1. Overall Accuracy: This metric is calculated as the ratio of correctly predicted instances (both true positives and true negatives) to the total instances.
2. Precision: Precision was calculated separately for Marfan and Non-Marfan classes.
3. Sensitivity (Recall): Sensitivity was calculated to gauge correct identification of Marfan and non-Marfan cases.
4. F1 Score: The F1 score is the harmonic mean of precision and recall. This metric provides a balance between precision and recall.
5. Area Under the ROC Curve (AUC): This metric assesses the ability of the model to distinguish between positive and negative cases (Fig. 2).
6. Confusion Matrix: A confusion matrix was generated to provide a detailed breakdown of the true positive, true negative, false positive, and false negative rates (Fig. 1).

Given that the total dataset comprised 672 images (182 Marfan images and 490 controls), the validation dataset comprised 134 images. In turn, these images were made up of 34 Marfan images and 100 control images. The confusion matrix for the model's predictions on the validation dataset are summarized in the following confusion matrix (Fig. 1).

Human Investigation Committee assessment (vertabim): “The Yale IRB determined that the investigator is not engaged in research involving human subjects. As such, IRB review and approval are not required.”

## 2 Results

The total number of Marfan patient images used in this study was 182. The total number of non-Marfan patient images used was 490.

Overall accuracy was 98.5 % (0 % false positive, 2 % false negative). Precision was 100 % for Marfan and 98 % for Non-Marfan. Sensitivity was 94 % for Marfan and 100 % for non-Marfan. See Table 1. See Confusion Matrix (Fig. 1.) F1 score was 97 % for Marfan facies and 99 % for non-Marfan facies. Area under the ROC curve was 100 % (Fig. 2.).

**Table 1:** Results of analysis.

Metric		Marfan	Non-Marfan
Overall accuracy	98.5 %		
False positive rate	0.0 %		
False negative rate	2.0 %		
Precision^a^		100 %	98 %
Sensitivity (recall)^b^		94 %	100 %
F1 score^c^		97 %	99 %

a Precision is the ratio of correctly predicted positive observations to the total predicted positives. Formulaically it can be expressed as: precision = true positives/(true positives + false positives). Precision indicates what proportion of positive identifications was actually correct. A high precision indicates a low rate of false positives.

b Sensitivity is the ratio of correctly predicted positive observations to all observations in the actual class. Formulaically it can be expressed as: sensitivity = true positives/(true positives + false negatives). Sensitivity measures how many of the actual positives the model captures through labeling it as positive. A high recall indicates a low rate of false negatives.

c The F1 Score is the weighted average of Precision and Recall. It takes both false positives and false negatives into account. Formulaically it can be expressed as: F1 = 2 × (precision x sensitivity)/(precision + sensitivity). The F1 score is useful when one would like to balance precision and recall. An F1 score reaches its best value at 1 (perfect precision and recall) and worst at 0.

## 3 Discussion

Diagnosis of Marfan Syndrome (MFS) traditionally leans on a combination of clinical evaluations and genetic testing, a process guided by the revised Ghent criteria. These include physical diagnosis, echocardiography, computerized tomography (CT), and genetic testing (in this era, usually via Whole Exome Sequencing (WES)). These components each play a vital role. Nonetheless, especially before WES or when WES is not available, uncertainty in precise diagnosis may prevail – a gap that the Convolutional Neural Network (CNN) model may help to fill, as an adjunct to traditional diagnostic methods. Also, this Artificial Intelligence technique may conceivably provide assistance in resolving the frequent conundrum represented by the frequent WES reports that a “Variant of Unknown. Significance (VUS) has been identified. A positive facial recognition test may suggest more likely veracity of the VUS.

The present study underscores the substantial potential of AI, specifically CNNs, to provide alternative supplementary approaches to the diagnosis for genetic disorders like Marfan Syndrome. This experience has shown that CNNs can offer an extremely accurate assessment. These results were achieved instantly and non-invasively upon application of the CNN program. The non-invasive nature of the CNN model and the speed and ease with which the testing can be performed can be expected to hold significant clinical value.

While the CNN model demonstrated impressive performance, it is crucial to address its limitations. The model's performance is contingent on the availability and quality of the input images, implying that varied lighting conditions and image quality could adversely affect diagnostic accuracy. Moreover, the model's reliance on observable facial features means that its utility may be limited in cases where the syndrome's facial manifestations are subtle or not yet fully apparent.

With respect to the collection of facial images for Marfan subjects, images were included only if it was clear from the context in which the image was found that the subject has been diagnosed with Marfan Syndrome. However, it is frequently not clear how the diagnosis was made. This is an area where extensions of this research may be improved.

It should also be noted that the images from which the Marfan subjects’ faces were excerpted were not originally intended for clinical application. Consequently, they include certain images with unusual orientation and facial expressions. However, the facial images for the non-Marfan group were also not collected for clinical application, and so exhibit similar shortcomings. As such, it is not expected that this should impact the reliability of the assessment of the model performance.

Additionally, it remains to be seen how well the model's performance would generalize across various racial groups. Although no racial groups were excluded from the dataset used for training and testing the model considered in this paper, the vast majority of images obtained for Marfan subjects were Caucasian. Although the non-Marfan subjects were more racially diverse, that dataset obtained was also majority Caucasian. In order to ensure that the model could be used reliably on all racial groups, it would be necessary to supplement the dataset with subjects from non-Caucasian racial groups.

In terms of overall perspective, due to lack of external/clinical validation and lack of patient characteristics, this manuscript should be considered as a preliminary pilot study whose utility in real-world application remains to be demonstrated.

Despite these limitations, the CNN model's benefits, including its high accuracy, speed, and cost-effectiveness, make it a promising tool for augmenting traditional diagnostic protocols for Marfan Syndrome. Future studies should focus on refining the model to handle varied image conditions and on exploring methods to identify subtle facial manifestations more effectively. At present, it has not been established which particular facial features are utilized by the CNN to make its determination of whether a particular subject has Marfan Syndrome.

*Future Directions*: While the results from this study are promising, further work is needed to fully realize the potential of CNNs in clinical practice. Future research should consider the real-world implementation of this CNN model, including the development of user-friendly interfaces for clinicians. Rigorous validation of the model on larger, more diverse datasets (in the dimensions of race and age, in particular) is also essential. Ethical considerations such as patient consent, data security, and privacy should be addressed before integrating this approach into routine healthcare practices.

It would also be valuable to address the shortcomings of the current research presented in this paper, including.

• *Improving the standardization of the images.* It may be valuable to collect images for training CNN models that make use of standardized facial orientation (or orientations), distance from the camera, picture resolution and facial obstructions (i.e. removing spectacles or goggles).
• *Ascertaining confirmed diagnosis of Marfan Syndrome.* As mentioned above, the subjects included in the Marfan group were self-reported as having been diagnosed with Marfan Syndrome. It would be valuable to compile a dataset which includes only subjects for whom their diagnosis, and the mode of their diagnosis, is known and certain.
• *Increasing the size of the dataset.* Although the size of the dataset used for training the model discussed in this paper is comparable to those for other medical studies, it is relatively small for computer-vision applications. This technique would likely perform better and be more reliable if the number of Marfan and non-Marfan subjects were to be increased materially.

In conclusion, this study provides an in-depth exploration of implementing a CNN model for the diagnosis of Marfan Syndrome using facial images. The analysis conducted shows that the CNN model can rival traditional diagnostic methods in terms of accuracy, while also offering advantages in speed and non-invasiveness. However, due to the limited and preliminary nature of this work, this should be viewed as only a pilot study. As AI continues to revolutionize healthcare, integrating such AI-based models into the diagnostic workflow could substantially enhance early detection and management of conditions like Marfan Syndrome. It may be possible to deploy such models to allow for screening in non-specialist settings, such as with general practitioners, nurses, schools and workplaces.

## Data availability statement

The data that support the findings of this study are available on request from the corresponding author.

## Funding

None.

## CRediT authorship contribution statement

**Danny Saksenberg:** Writing – original draft, Formal analysis, Conceptualization. **Sandip Mukherjee:** Writing – review & editing, Project administration, Conceptualization. **Mohammad A. Zafar:** Writing – review & editing, Data curation. **Bulat Ziganshin:** Writing – review & editing, Data curation. **John A. Elefteriades:** Writing – review & editing, Writing – original draft, Project administration.

## Declaration of competing interest

The authors declare the following financial interests/personal relationships which may be considered as potential competing interests:John A Elefteriades reports a relationship with Coolspine that includes: equity or stocks. If there are other authors, they declare that they have no known competing financial interests or personal relationships that could have appeared to influence the work reported in this paper.
